# Postural Sway in Older Patients with Sagittal Imbalance and Young Adults during Local Vibratory Proprioceptive Stimulation

**DOI:** 10.3390/healthcare9020210

**Published:** 2021-02-15

**Authors:** Tadashi Ito, Yoshihito Sakai, Kazunori Yamazaki, Yohei Ito, Keitaro Kawai, Yoshiji Kato, Hideshi Sugiura, Yoshifumi Morita

**Affiliations:** 1 Three-Dimensional Motion Analysis Room, Aichi Prefectural Mikawa Aoitori Medical and Rehabilitation Center for Developmental Disabilities, Okazaki 444-0002, Japan; 2 Department of Physical Therapy, Graduate School of Medicine, Nagoya University, Nagoya 461-8673, Japan; hsugiura@met.nagoya-u.ac.jp; 3 Department of Orthopedic Surgery, National Center for Geriatrics and Gerontology, Obu 474-8501, Japan; jsakai@ncgg.go.jp; 4 School of Health Sciences, Faculty of Clinical Engineering, Fujita Health University, Toyoake 470-1192, Japan; ymzkk@fujita-hu.ac.jp; 5 Department of Electrical and Mechanical Engineering, Graduate School of Engineering, Nagoya Institute of Technology, Nagoya 466-8555, Japan; y.ito.359@nitech.jp (Y.I.); k.kawai.750@nitech.jp (K.K.); morita@nitech.ac.jp (Y.M.); 6 Department of Physical Therapy, Nagoya Heisei College of Nursing & Medical Care, Nagoya 464-0850, Japan; yoshi-waterfalls@cam.hi-ho.ne.jp

**Keywords:** proprioceptive, postural control, sagittal imbalance, muscle spindle

## Abstract

This study aimed to assess differences in somatosensory control strategies between older patients with sagittal imbalance and young adults during postural tasks. The center of pressure displacement in 27 older patients with sagittal imbalance and 27 young adults was determined upon standing blindfolded on a balance board. Vibratory stimulation at 56 to 100 Hz was applied bilaterally to the gastrocnemius and soleus muscles (GS) and lumbar multifidus to evaluate the contributions of proprioceptive signals to postural control. Data of older patients and young adults were compared using the Mann–Whitney U-test or independent sample t-tests. Compared with the young adults, the older patients were significantly more reliant on the GS (*p* < 0.005) for their postural control and showed a higher relative proprioceptive weighting ratio (RPW) (*p* = 0.038). The postural strategy adopted by the older patients depended on the level of proprioceptive stimulation applied to the GS, and the postural control strategy of the ankle correlated with RPW. Overall, this study identifies RPW as a novel measure of postural strategy in older patients with sagittal imbalance and provides an understanding of strategies used to maintain balance, which may assist in developing preventative measures to reduce the risk of falls.

## 1. Introduction

Postural stability is essential for all activities of daily living. However, individuals may adopt different strategies for achieving postural stability, as it depends on the integration of the spine and sensorimotor systems [1]. In a previous study, we found that proprioceptive cues in healthy young adults were principally required for orientation of postural control rather than for control of upright posture, for which vestibular and visual cues were more important [2]. Diseases affecting postural alignment of the spine may lead to decreased quality of life, weakened back muscles, and postural instability [3,4,5,6]. Sagittal balance is a mechanical system consisting of osteoarticular elements in the spine–pelvis structure of the lower legs. It allows a direct correlation of the postural balance as a predictive and prognostic factor of lumbar degenerative diseases [7]. The degenerative process primarily affects the lumbar spine and involves intervertebral ligaments, bone, facet joints, and discs [8]. Moreover, spinal alignment is a unique anatomical feature, which allows the maintenance of a neutral upright posture with minimum energy expenditure [8]. Other researchers reported that older adults with spinal deformity have an impaired perception of upright vertical alignment that worsens with age-related impairment of proprioceptive and vibratory input from the lower extremities [9]. Correct posture allows one to retain a postural balance and limits movement in relation to the support plane, ensuring postural stability with minimum muscle involvement [10,11]. Recent studies have reported that sagittal balance and spinopelvic alignment contributed to an energy efficiency posture of people in healthy or diseased states [12,13]. Moreover, stabilizing the spinal alignment is important for maintaining a correct posture [14]. When undertaking postural control tasks, adolescents with idiopathic scoliosis experienced difficulty in reweighting proprioceptive inputs following brief periods of proprioceptive deprivation [15]. Other studies in adult patients with spinal deformities have reported postural instability when standing [16,17]. Those results may indicate that patients with sagittal imbalance might have poor proprioception. Thus, proprioception is essential for maintaining optimal spinal alignment and coordinating muscle activation [18].

Postural tasks during local vibratory stimulation have become important clinical tools for assessing proprioception in adults with various disorders, including spinal deformity, low back pain (LBP), lumbar spondylosis, and non-specific LBP [19,20,21,22]. Postural task analyses have shown that healthy young adults and older patients depend on proprioceptive inputs from the trunk and lower leg regions [23,24,25,26,27]. A previous study identified age-induced alterations in muscle spindles by investigating the differences in the postural control strategies of the ankle and hip between young adults and older adults [28,29].

Postural instability has previously been observed in patients with LBP, along with decreases in the relative proprioceptive weighting ratio (RPW) at muscle spindles in the trunk [20,21]. RPW provides additional information about the proprioceptive dominance; a high value of the RPW indicates perfect reliance on lower limb input (“lower limb-focused strategy”), whereas a low value of the RPW indicates perfect reliance on trunk input [19,30]. Given that the postural control in older patients with sagittal imbalance depends on proprioceptive inputs, examining the RPW in the postural sway of the ankle and hip during local vibratory stimulation scenarios may provide valuable insights. Moreover, the use of local vibratory stimulation to assess the posture of patients with sagittal imbalance due to spinal deformity in older adults may assist in the evaluation of postural instability in such patients and provide valuable functional assessments in those with poor proprioception. Furthermore, since postural control strategies for older patients with sagittal imbalance depend on proprioceptive inputs, the effect of local vibratory stimulation on the postural sway of the ankle and hip may provide valuable information. However, the difference of RPW in older adults with sagittal imbalance and young adults is not yet clearly understood.

The purpose of this study was to assess postural stability in patients with sagittal imbalance using local vibratory stimulation. Our aims were to compare postural stability between patients with sagittal imbalance and young adult controls and determine the differences in postural sway of the ankle and hip in patients with sagittal imbalance. We hypothesized that patients with sagittal imbalance would have poor proprioceptive inputs from the trunk compared to healthy controls. In addition, we aimed to investigate the relationship between sagittal alignment and proprioception control strategies on ankle and trunk in patients with sagittal imbalance.

## 2. Materials and Methods

### 2.1. Participants

Individuals with sagittal imbalance and aged between 65 and 83 years were eligible for this study. The participants were enrolled between December 2018 and September 2020 after visiting the authors’ institute or the Nagoya Heisei College of Nursing & Medical Care, or responding to a call for volunteers, or for other reasons. Twenty-seven older adults with spinal column stenosis and spondylitis deformans who presented for conservative treatment were recruited for the study. A diagnosis of lumbar spondylosis was confirmed using L1/2 to L4/5 area magnetic resonance imaging by a spine surgeon (Y.S.).

The participants had medically diagnosed LBP but had no arthralgia and did not require assistance in maintaining a standing posture. The diagnosis of LBP was confirmed by a spine surgeon. For control purposes, 27 healthy young adults aged over 18 years and sex-matched with the older cohort were recruited. None of the participants required assistance in daily living activities. Participants with the following characteristics were excluded: vestibular function disorders, spinal compression fracture, spinal cord tumor, spinal infection, paralysis, ataxia, neurological disorders, balance disorders, or a history of spinal surgery.

### 2.2. Postural Control Assessment

Center of pressure (COP) displacement was recorded using a balance board (Wii; Nintendo Co., Ltd., Kyoto, Japan) [31,32,33]. Balance board data were acquired using a sampling frequency of 100 Hz and calculated using MATLAB (MathWorks, Inc., Natick, MA, USA). Participants wore an eye mask and stood barefoot on the balance board with their feet together. They were instructed to remain still and relaxed with arms hung loosely at the side. Each participant performed three trials of postural tasks: one with no vibratory stimulation, one with vibratory stimulation of gastrocnemius and soleus muscles (GS), and one with vibratory stimulation of lumbar multifidus (LM). In order to evaluate postural sway during proprioceptive inputs to the muscle spindles, the response frequency ranging from 56 to 100 Hz was analyzed. For information regarding proprioceptive dominance, RPW was calculated as follows: RPW [%] = (RMS_GS_)/(RMS_GS_ + RMS_LM_) × 100), where RMS_GS_ and RMS_LM_ are the root mean square of the COP displacement in the anteroposterior direction during GS and LM vibrations, respectively [6,28,34,35]. RMS was calculated as follows:

$${\mathrm{RMS}}_{*}=\sqrt{\frac{1}{1500}\overset{3000}{\underset{n=1500}{\sum }}{\left\{Y_{*}^{\mathrm{Dur}}\left(n\right)-{\bar{Y}}_{*}^{\mathrm{Pre}}\right\}}^{2}}$$

where n is the number of data series; Y_*_^Dur^(n) is the CoP in the anteroposterior direction in the duration section (Dur-section); Y_*_^Pre^ is the mean value of CoP in the anteroposterior direction in the pre-sectiion (Pre-section); the subscript “*” is used to distinguish the stimulation body locations, namely, the GS or LM.

### 2.3. Muscle Vibration

**Figure 1:**
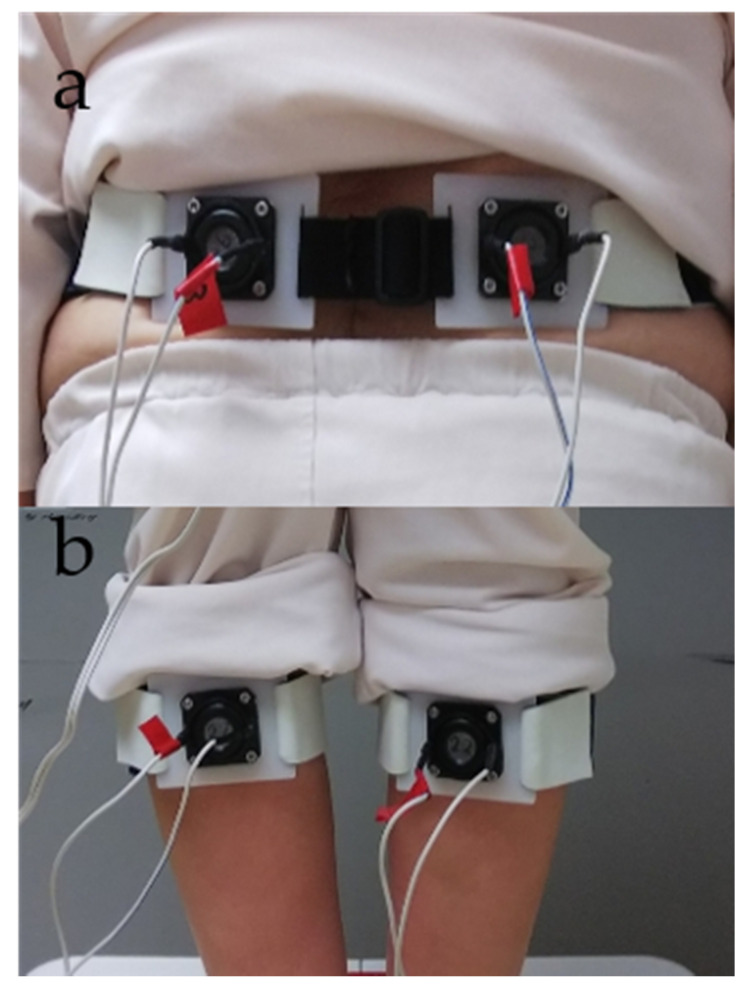
Vibration device setup. (**a**) Lumbar multifidus (LM). (**b**) Gastrocnemius and soleus (GS) muscles.

Vibratory stimulation was applied alternately by fixing vibrators from the vibration device on the participant’s GS and LM (Figure 1).

A sweep frequency [34] was used to deliver the vibration, which was continuously changed from 56 to 100 Hz (frequency ascend mode) or 100 to 56 Hz (frequency descend mode) for 15 s. The ascend or descend sweep frequency mode was randomly determined for each subject [34]. The measurement procedure entailed application of the vibration to both the GS and LM. The applying order to GS or LM was randomly determined for each subject. Each application required 30 s; this time was divided into the first 15 s and last 15 s to form the pre-section and duration section, respectively. In the duration section, the vibratory stimulus was applied to the GS or LM of the participant with his/her eyes closed [6,19,27,28,34,35]. The number of repetitions of stimulation was two, and the stimulation was given to GS and LM once for 15 s. Therefore, the total stimulation time was 30 s. In addition, the participants rested on a chair once for 60 s between measurements. In previous studies, the task has been performed once because there was an influence of getting used to the postural sway test [6,19,20,21,27,28,34,35]. Therefore, in this study, the task was performed once as well. Assessments were performed by an experienced research assistant, a physiotherapist, and a physician.

The steps of this experiment were described as follows:

1. Measurement of postural sway during stimulation to GS or LM (30 s)
2. A sitting rest (60 s)
3. Measurement of postural sway during stimulation to GS or LM (30 s).

### 2.4. Low Back Pain Assessment

Pain was assessed using the visual analog scale (VAS) (0–10) [6,19]. All participants were asked to complete a pain questionnaire.

### 2.5. Sagittal Imbalance

Sagittal vertical axis (SVA) has been proposed as a criterion for sagittal alignment [7] and was defined in our study as the horizontal offset from the posterosuperior corner of S1 to the vertebral body of C7. SVA was measured using a SYNAPSE (Fujifilm Medical Co., Ltd., Tokyo, Japan). Sagittal imbalance was defined as SVA >40°. SVA was measured in only the patient group.

### 2.6. Statistical Analysis

Normal distributions were confirmed using the Shapiro–Wilk test. Data of older and healthy young adults were compared using the Mann–Whitney U-test or independent sample t-tests. Moreover, Spearman’s rank correlation analysis was performed to determine the relationship between SVA and COP excursion of GS and LM. Effect sizes were calculated using r or Cramer’s V. Effect sizes with r = 0.1 or r = −0.1 were considered small; those with r = 0.3 or r = −0.3, moderate; and those with r = 0.5 or r = −0.5, large. All data were analyzed using IBM^®^ SPSS^®^ Statistics for Windows version 24.0 (IBM Corp., Armonk, NY, USA). Statistical significance was considered when the *p*-value was <0.05.

### 2.7. Sample Size

The sample size for the Mann–Whitney U-test was determined using power analysis. Power analysis was performed with G*Power (Heinrich Heine University, Düsseldorf, Germany) using an alpha of 0.05, a power of 0.80, and a large effect size (d = 0.8) for a two-tailed test [36,37]. Based on these assumptions, the required sample size was calculated as 54 (27 older patients with sagittal imbalance and 27 young adults).

## 3. Results

**Figure 2:**
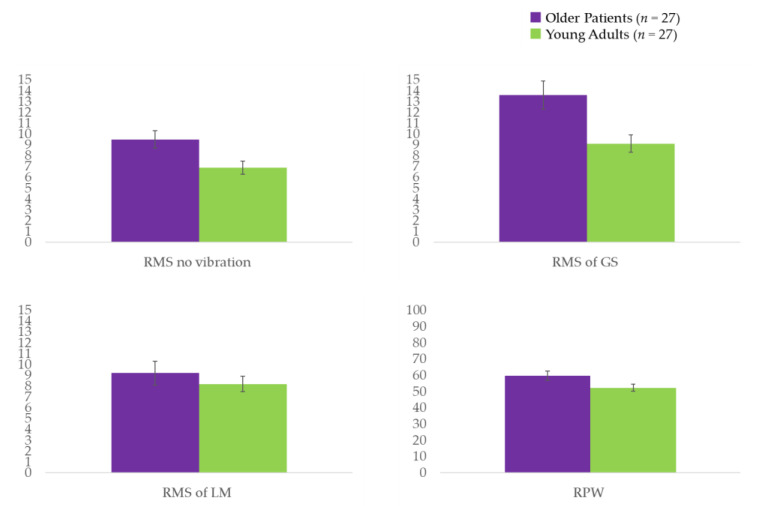
RMS values of the COP displacement for the trials on the balance board. COP: center of pressure; GS: gastrocnemius and soleus muscles; LM: lumbar multifidus; RMS: root mean square; RPW: relative proprioceptive weighting ratio. A unit of RMS = mm; a unit of RPW = %.

For the older patients with sagittal imbalance, the SVA range was 44.1–178.2, with an average of 93.1 ± 35.2. Except for LBP (VAS), mean age, and height, there were no significant differences in participant characteristics between the older patients with sagittal imbalance and the healthy young adults (Table 1). A larger postural sway was observed when analyzing COP of no vibratory stimulation in the older patients compared to that in the healthy young adults (*p* = 0.023) (Table 2 and Figure 2). Older patients also displayed a significantly larger postural sway during the application of vibrations to the GS (*p* < 0.005) (Table 2 and Figure 2), as well as a nonsignificant trend for a larger postural sway when local vibrations were applied to the LM. A dependence on ankle strategy was observed in the older patients compared to that in the healthy young adults based on the RPW (*p* = 0.038) (Table 2 and Figure 2). Furthermore, results of the Spearman’s rank correlation analysis showed that in older patients, the response of the LM vibration was not significantly correlated with SVA (r = −0.294; *p* = 0.136), whereas the GS had a moderately negative correlation with SVA (r = −0.413; *p* = 0.032).

## 4. Discussion

This is the first study to examine postural control strategies in older patients with sagittal imbalance using proprioceptive stimulation. Our findings demonstrate that such patients, when standing, have decreased reliance on proprioceptive signals from the trunk with a 56 to 100 Hz vibratory stimulus. There was more reliance on an ankle strategy to maintain stability compared to the young adult controls. A previous study showed a significant decrease in proprioceptive sensation among patients with spinal imbalance for the trials with eyes closed [38]. Although patients with poor postural control may appear balanced when using an ankle strategy under static conditions, reduced proprioceptive signaling of the trunk may exacerbate underlying postural instability and induce a fall. Sensitivity to the control in the COP displacement is caused by an increase in tension of the paraspinal muscle [39,40]. Previous studies have suggested that patients with spinal deformities may maximally exert compensatory muscles and postural reserves when using strategies to maintain postural control [22]. In our cohort, postural control was achieved with ankle strategies both with and without vibratory stimulation. These results suggest that postural control is impaired regardless of the proprioceptive input during local vibratory stimulation. This pattern of impairment is associated with significant limitations to daily living [41]. In our patients, postural instability was indicated by decreased muscle spindles in the trunk and postural control without vibratory stimulation. Therefore, the difficulties associated with postural sway may be caused by greater ankle movements due to an over-dependence on proprioceptive inputs as well as poor balance function.

As a component of motor control, proprioception plays a substantial role in postural stability [42,43]. Muscle spindles in the triceps surae are typically in an elongated position, which increases their sensitivity when standing with the heels on the ground [44]. Postural control that is mainly based on proprioceptive input from the ankle benefits from a similar change in position [21]. In addition, local muscle vibration can excite muscle spindles and increase the muscle firing rate [45]. There were studies that supported the notion that proprioceptive function (alterations in postural control and decrease of muscle spindles number in paravertebral muscles) is impaired in patients with sagittal imbalance [46,47]. Thus, it has been speculated that proprioception was involved in the control of stability of the spine, with muscle spindles acting as a regulatory feedback mechanism [48,49]. Therefore, an interaction between muscle spindles and simultaneous muscle activation may exist during proprioceptive control strategies in older patients with sagittal imbalance. Postural instability in such patients parallels findings on proprioception in adults with spinal deformity [22]. Previous studies have also demonstrated persistent alterations in standing static balance after radiographic correction of these deformities, suggesting a sensorimotor contribution to reduced postural stability despite postural realignment [16]. As a result of limitations in trunk motion due to poor proprioception among older patients with sagittal imbalance, larger compensatory motion from the ankle is required to correct posture when standing upright. This may occur because such patients tend to implement ankle strategies to maintain balance. Furthermore, poor proprioception affecting the spinal alignment will always have a negative effect on posture and balance control, leading to postural disorders in response to the changes in muscle tension.

Our findings suggest that differences in proprioceptive control strategies between older patients with sagittal imbalance and young adults are more detectable when the eyes are closed. In addition, the postural task in the RPW and RMS differentiates better the balance behaviors between older patients with sagittal imbalance and the younger participants. This may be due to differences in balance control and proprioceptive input of the ankle and trunk. Further investigation is required to understand the association between vibratory stimulation and changes in proprioceptive control strategies.

Furthermore, our study’s novel finding was the significant relationship between postural control of the GS muscle and SVA in older adults with sagittal imbalance. Our data suggested that the COP displacement of the GS at the proprioception decreased as SVA increased. Drzał-Grabiec et al. reported a correlation demonstrating that the body posture and the spinal alignment had an impact on the response from the balance [50]. In addition, a previous study reported that changing the angle of kyphosis affected the dorsal and calf muscle tension, contributing to the weakening of equivalent reactions [51]. In other studies, it was reported that the mechanism that controls the stability of a complex structure, such as the spine, also suggests the involvement of proprioception and, specifically, muscle spindles as a regulatory feedback mechanism [48,49]. Thus, the dependence on the proprioceptive strategy of the lower limbs in older adults with sagittal imbalance could be associated with a decrease in SVA. Further, increased SVA decreases the dependence of proprioception reaction on lower limbs controls through a change in postural muscle tension. This disproportion may indicate that sagittal imbalance also affects other mechanisms related to the control of COP displacement and suggests that further study of interrelationships is warranted in the future.

The limitations of our study include the fact that we only focused on GS and LM vibration, and our conclusions may not be generalized for the vibration of other muscles. In addition, the difference in proprioceptive inputs with respect to the healthy older adults and patients was not evaluated in this study. Assessing this could provide more information on the possible influence of SVA. Furthermore, the significant differences in age between the two cohorts in our study may warrant investigation of proprioceptive control strategies due to vibration among older patients of similar age.

## 5. Conclusions

Older patients with sagittal imbalance appear to demonstrate postural instability and decreased trunk proprioceptive input compared with sex-matched younger controls. Changes in GS sway were significantly greater in older patients with sagittal imbalance than those in young adults. In addition, the older patients showed heavier reliance on ankle strategies for maintaining balance when subjected to vibratory stimulation. These findings suggest that abnormal postural alignment in older patients with sagittal imbalance contributes to an impairment in postural stability with poor proprioception on the trunk. Understanding the strategies used by such patients to maintain balance may assist in the development of preventative measures to reduce their risk of falls.

## Figures and Tables

**Table 1:** Participant characteristics.*

Variable	Older Patients (*n* = 27)	Young Adults (*n* = 27)	Effect Size (*r* or Cramer’s V)	*p*-Value
Age (years)	78.0 (65–83)	20.0 (18–28)	−0.9	0.001
Sex (male/female)	15/12	15/12	0.0001	1.000
Height (cm)	155.7 (6.8)	165.2 (6.8)	0.5	0.0001
Weight (kg)	58.0 (31.6–79.4)	60.2 (41.7–77.9)	0.1	0.562
BMI (kg/m^2^)	23.9 (4.3)	22.1 (2.4)	0.3	0.059
VAS (cm)	6.7 (0–10)	0 (0–2.7)	−0.9	0.0001

* Data are presented as mean (standard deviation) or median values (range). The *p*-values for age and weight were determined using the Mann–Whitney U-test; the remaining *p*-values were determined using the independent t-test or chi-square test. BMI: body mass index; VAS, visual analog scale.

**Table 2:** Displacement of the COP during local vibratory stimulation in young adults and older individuals standing on a balance board.*

	Older Patients (*n* = 27)	Young Adults (*n* = 27)	Effect Size (*r*)	*p*-Value
RMS no vibration (mm)	8.4 (2.8–18.1)	6.2 (3.1–16.4)	−0.3	0.023
RMS_GS_ (mm)	13.3 (5.0–24.3)	8.9 (2.9–21.6)	−0.4	<0.005
RMS_LM_ (mm)	8.4 (3.2–24.3)	7.7 (3.3–15.4)	−0.04	0.789
RPW (%)	59.8 (11.1)	52.3 (14.4)	0.3	0.038

* Data are presented as the mean (standard deviation) or median values (range). The *p*-values were determined using the Mann–Whitney U-test or the independent t-test (RPW). COP: center of pressure; GS: gastrocnemius and soleus muscles; LM: lumbar multifidus; RMS: root mean square; RPW: relative proprioceptive weighting ratio.

## Data Availability

All of the relevant data are presented within the manuscript. All data are available from the authors on request.
